# Food Safety Governance in the Age of COVID-19: How Does Employees’ Attitude on Public-Private Governance System Affect Their Willingness to Blow the Whistle on Food Violations?

**DOI:** 10.3390/healthcare11020167

**Published:** 2023-01-05

**Authors:** Zechen Liu, Zengjin Liu, Guanghua Han

**Affiliations:** 1 School of International and Public Affairs, Shanghai Jiao Tong University, Shanghai 200030, China; 2 Shanghai Academy of Agricultural Sciences, Shanghai 201106, China

**Keywords:** food safety, whistleblowing, post-pandemic, private-public governance, restaurants violation

## Abstract

Online food delivery increases dramatically during the COVID-19 era and has grown into a global marketplace worth more than $150 billion dollars, necessitating a more effective and responsive governance system. Public-private governance systems with whistleblowing to the public are seen as an effective tool for addressing the formidable challenges of food security in modern society. Accordingly, this study aims to explore the determinants of whistleblowing intentions and to propose policy policies for the whistleblowing system to fully utilize the advantages of public-private governance systems. Through empirical research, this paper finds that employees’ perceived effectiveness of government authorities, as well as their familiarity with whistleblowing systems, positively affect their intentions to blow the whistle. However, the whistleblowing intent of restaurant employees is adversely affected by the online platform’s focus. The root cause lies in the employee’s trust in both the government sector and corporate sector. This study thus argues that a suite of measures to promote individual trust in public-private governance systems is desired and that this is an effective means of better mitigating food safety governance challenges in terms of capacity and resources.

## 1. Introduction

The rapid global spread of COVID-19 over the past two additional years has already become the most severe pandemic of infectious diseases since the 1918 influenza worldwide. The most well-known and prevalent route of viral transmission is human-to-human contact through respiratory droplets, but compared to the influenza A virus, SARS-CoV-2 is able to survive on human skin for up to 9 h, which may increase the potential for transmission through contact and thus the spread of the outbreak [1]. Studies have shown that the new coronavirus is able to attach to the food itself and its packaging for a longer period of time without losing its high infectivity. The consumption of contaminated food surfaces, or in the packaging of food in an infected room either by handling food without precautions or by sharing food with an infected individual, can result in exposure to the novel coronavirus [2,3,4], especially in restaurants, manufacturing companies and in the community. It was reported that physical contact and food sharing during the Singapore conference in January 2020 led to multiple cases of COVID-19 [5]. The seriousness of the spread of COVID-19 through contaminated surfaces has been noted by both the government and the general public alike and are paying increasing attention to food safety issues because of the fundamental role food plays in daily life. Concerns have been raised about the potential for food to become contaminated with viruses during production, processing, packaging, and transportation. For example, if a COVID-19 patient coughs, sneezes, speaks, or breathes, they will shed liquid particles that are carriers of the virus. Infected droplets and aerosols may land on surfaces and objects that are frequently touched or may be directly inhaled by other individuals who are standing nearby [6]. As long as the object provides an environment favorable to the virus, the virus can remain highly stable on its surface [7]. Whether it is raw materials or all aspects of distribution, it can be said that how to ensure food safety is not only an issue faced by the current industry but also the focus of consumer attention. As a result, food safety issues provoke more attention and anxiety in the COVID-19 age.

The guidelines are suggested by international organizations and many countries in the food industry due to the riskiness of food production and transportation [8]. For example, World Health Organization suggests several key actions to remain food free of virus contamination that includes improved sanitation and clean-up measures, strengthening protocols, and sanitization of high touch points (WHO, 2020) [9,10]. The U.S.-based company, the Food and Drug Administration, provides guidance to industry members and consumers on the safety of food during COVID-19. In the EU, food safety legislation obliges all food business operators to ensure that all employees take steps to ensure hygiene (EU, 2020) [11].

In 2020, following the interruption in the transmission of the local outbreak in China, numerous local aggregated outbreaks of COVID-19 occurred sequentially in various locations. The on-site epidemiological investigation and analysis allowed experts at the China Center for Disease Control and Prevention (CDC) and provincial and municipal CDCs to quickly determine the source of 7 locally aggregated epidemics, molecular tracing of the COVID-19 viral genome, as well as dynamic virus detection. The outbreaks were confirmed to be caused by imported cold chain products contaminated with neo-coronavirus resulting in the infection of employees in the cold chain, which solidified scientific evidence for the cold chain as a vehicle for the transmission of COVID-19 viruses. In addition, the International Union of Food Science and Technology (IUFoST) and the Chinese Institute of Food Science and Technology (CIFST) believed that the food industry is facing many challenges, with consumers having doubts about whether raw food or food packaging can transmit new coronaviruses, followed by cleaning and disinfection issues in food manufacturing, retail, and restaurants, which undoubtedly place higher demands on the food industry in terms of strengthening food safety management and improving hygiene and cleanliness.

While the economies could be rebuilt from the COVID-19 pandemic, the social impacts will endure for a long period of time and fundamentally highlight the need for improved governance. However, the government is often constrained by the cost of oversight (e.g., manpower, money, time, etc.) and cannot oversee the entire process at all times. Take-away food is an important form of food and drinks consumption in many cities, making government regulation more difficult. As a result, take-away food marketers have shown the characteristics of large quantity, small scale, wide dispersion, and uneven levels of quality, which undoubtedly cut across the regional boundaries of local regulation and pose risks in multiple ways in the age of epidemics. First, the geographic location of restaurant patrons extends further, so that the food supply chain often spans multiple regulatory regions, and the spillover effects of risk are greater than in traditional settings. Second, as well as the wide range of movements of food delivery staff and a large number of people in contact with them (including restaurant stores and consumers), risks can be rapidly transmitted. Third, takeaway foods are open for business day and night, but on-site sampling and inspection cannot be conducted at all times. Consequently, during an epidemic, takeaway foods face greater safety implications and greater difficulty in monitoring food safety.

In fact, scholars have been engaged in the study of food safety governance since the beginning of this century, produced notable results, and formed a more mature theory system. Julie A. Caswell concluded that food safety regulation in the developed world has a greater focus on ex-ante regulation and will place a greater emphasis on the importance of prevention, as more developing countries focus on regulation during and after events [12]. Henson was the first (2001) to propose the introduction of a system of public-private collaboration in the governance of food security [13]. Cooperation between public and private governance is the essence of this system, and cooperative regulation is a form of cooperation aimed at achieving public management. One of the most characteristic features of collaborative public-private governance is that private subjects are engaged in self-discipline or self-regulation, i.e., groups or individuals autonomously assume the social responsibility to achieve public ends while pursuing their own self-interest, on the basis of their status as a basic subject. Martinez et al. also argue that collaborative governance of food safety can compensate for the inadequacies of government regulation in order to simultaneously reduce the cost of governance and enhance regulatory efficiency [14]. Li Qin suggests that the effectiveness of cooperation between the government, the market, and third-party organizations should not be overlooked, and that food security should be governed jointly with other stakeholders [15]. Garcia conducted a study on shared governance models for food security, comparing UK and US governance models, suggesting a lack of resources and a conflict of interest between the various ministries, and that government departments and third-party organizations should work closely together to effectively avoid wasting resources, allocate resources rationally and improve the efficiency of governance [16]. Rouvière et al. (2012) constructed a conceptual framework for the implementation of co-regulatory governance of food safety and applied it to an imported supermarket in Perpignan, France, favorably improving the quality of the imported food [14].

The concept of social co-governance has developed into an important component of food safety governance in developed economies and has contributed favorably to the solution of their food safety problems. The concept of “social governance” was not introduced in China until 2013 and has not been around long enough for public involvement in food security governance to be explored. Much of the research on collaborative public-private governance has focused on summaries of overseas experiences and the willingness of residents to participate and third-party interventions [17,18], with the belief that residents play an important role as one of the key participants in food safety governance [19], but there has been little research on the factors influencing whistleblowing on Food Violations.

A large number of studies conducted by academics from a variety of countries have found that information disclosure, food safety training, improved store qualification review mechanisms, and third-party supervision have all been shown to be effective methods of managing food safety risk [20,21,22]. Whistleblowers, as direct participants in the production of food safety, have a natural advantage in identifying food safety issues in the first place. Internal and external monitoring networks, if they can be combined, can compensate for the lack of, and high cost of, government surveillance. For example, the involvement of food industry insiders in food safety reporting may also serve to promote social control and restrict operators from regulating their activities. In the United States, for example, the Whistleblower Protection Act, as well as the U.S. Food Safety Modernization Act encourages the public, including company employees, to appropriately report food safety issues around them. Furthermore, the EU is adept at mobilizing social governance forces for food risk management, drawing on public participation and acceptance to develop risk policies. China’s traditional culture, however, can present barriers to food safety reporting. On the one hand, traditional Chinese Confucian culture fosters individual contribution to society in terms of value orientation, whereas on the other hand viewing reporting as unethical and undesirable personal behavior. For example, are restaurant employees prepared to report food safety violations in restaurants when the government and regulatory authorities are not in a position to properly control food safety risks? Therefore, this paper investigates the determinants of employees’ willingness to report food security violations.

Collaborative governance of food safety has clearly become a consensus across all sectors of society, and there is an abundance of research that provides a good foundation for this paper. However, much of the existing research on whistleblowing in food safety breaches focuses on policy design and associated qualitative analysis. Little empirical research has been conducted on the influencing factors of whistleblowing. As a result, it is difficult to integrate existing theoretical research into practice and lacks empirical support. Therefore, the purpose of this paper is to propose a framework for analyzing the factors that influence employees’ willingness to participate in public-private partnerships for food safety by whistling food safety violations. This validates the theoretical framework, which is intended to facilitate public-private partnerships.

## 2. Theoretical Foundation and Hypothesis

How to scientifically guide individuals to actively make food safety reports, effectively completing the private part of the collaborative public-private governance piece, is a key breakthrough in addressing the socialized problem of food security to form the social governance norm for food safety. Willingness is a significant predictor of behavior and is the entry point for understanding behavior. It is therefore important to explore factors influencing employees’ willingness to blow the whistle on food violations, which help to develop the collaborative public-private food safety governance.

**Figure 1:**
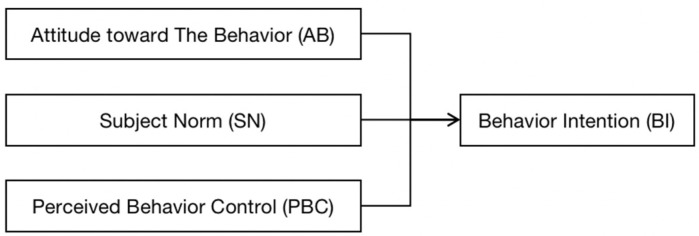
Framework of employees’ planned behavior.

According to the theory of planned behavior (TPB), people’s attitude toward the behavior (BA), subject norms (SN), and perceived behavioral control (PBC) jointly affect the intention (BI) of the behavior, which is thought to directly affect the behavior [23] (Figure 1). At the same time, the opportunity to take action is affected positively by people’s intentions [24]. Reporting behaviors are often not readily observable, making it difficult to measure actual reporting and the complex relationship between the investigation of misconduct in the workplace through direct observation. The best way to do this is to study real-world examples of whistleblowing behaviors and behaviors, but researchers remain limited to examining reporting intent when studying whistleblowing behaviors, in part due to their casual nature [25,26]. Whistleblowing is a sensitive topic and as such previous research has often used intention to report as a proxy for actual whistleblowing behavior [27], and this paper considers intention to report as a subject of study. Among restaurant staff, their reporting behavior regarding takeaway food risks is also influenced by their attitudes, social management, and capacity to make reports. Restaurants in particular face a great deal of pressure to report, including loss of work, potential personal injury, loss of income, and pressure from colleagues.

Meanwhile, restaurant employees possibly face reduced wages, loss of benefits, and other losses when food safety risks are discovered through government inspections and the imposition of fines [28,29]. Consequently, when the restaurant’s level of food security is lower than expected, the restaurant employee may have the attitude to change the level of food safety and may be willing to change the status quo by making a report. The management and education provided by the government or online platform to the restaurant at this stage is a reflection of the importance that the government or platform places on the safety of food in the restaurant. Staff may therefore be fully aware that their willingness to change the status quo and potential whistleblowing behavior will be supported by the government or the platform itself. As a result, the government or platform’s management and education of the restaurant is the primary normative factor for employees to report violations of food safety. Of course, employees’ food safety awareness and understanding of the reporting system is also a reflection of their perceived ability to exert control over their behavior. These capabilities help employees quickly identify food safety violations that need to be reported, as well as identify the proper channels and evidence to report.

### 2.1. The Perceived Effectiveness of the Public-Private Governance System

Restaurants that manage O2O takeaway food, i.e., online ordering and offline delivery, are managed both by government authorities and by online platforms. Government authorities have statutory obligations to regulate food safety issues and to deter violators of the laws. Government regulation of private sections is normally referred to as public governance whereas private section regulation is normally considered private governance. Online platforms are portal interfaces between restaurants and customers, which manage the food safety of restaurants in order to gain market share and competitiveness. As a result, online platforms provide incentives to take private governance of restaurants’ food security. One of the largest four online food delivery platforms in the U.S., Uber Eats, for example, only allows restaurants with 2 food hygiene scores to be listed on its platform. In addition, Uber Easts undergo regular inspections of restaurants that are subject to certain requirements [30]. As a result, restaurants are under collaborative public-private governance.

According to the theory of planned behavior, the individual’s behavioral intention is determined in part by their attitudes toward the behavior [31,32]. The employees, who have a positive attitude towards the efficacy of collaborative public-private governance, become more active in the public-private collaborative governance system and thereby is manifested in the whistler blowing of food safety violations. There are two ways in which collaborative public-private governance performance provides individuals with the confidence to participate in governance. Effective collaborative governance between the public and private sectors demonstrates trust and credibility, which are essential for public participation. In contrast, the good performance of the governance system means that it demonstrates competence and integrity to whistleblowers in terms of protecting personal information and providing effective interventions. The whistleblower’s greatest concern is the disclosure of personal information, which may result in potential personal harm, loss of social capital, and loss of job opportunities. It is difficult to obtain evidence of violations, and when a whistleblower takes the risk of making a report, he or she expects the regulator to act promptly and efficiently. This reduces the incentive for whistleblowers to coordinate with government agencies when they believe regulators will not effectively investigate and prosecute violations [33]. Therefore, we have Hypothesis 1.

**H1.**  *Perceived effectiveness of the public-private governance system positively affects employees’ intentions to blow the whistle on food safety violations.*

### 2.2. Guidance from the Public-Private Governance System

Guidance from public-private governance systems corresponds to subjective norms in the theory of planned behavior. Given the specialized nature of food safety and the fact that unsafe production raises the potential for serious risks to public health, the government and platform have guidelines (including normative training, education, etc.) for the operation of restaurants. Internalization of behavioral norms theory holds that good education can embed norms and beliefs in the behavior system of the educated person (Aronson 1969) [34]. Thus, restaurant guidance from regulatory agencies can educate operations managers about the rules that should be followed in food production and the penalties that come with breaking the law. In fact, studies have shown that individuals with experience in food safety education will have more relevant knowledge and improved behavior [31]. Since food safety is related to public health, there are usually severe legal penalties for illegal acts. For instance, the U.S. Criminal Fine Enforcement Act 1994 applies to fines related to food security scandals. At the same time, the FDA’s criminal investigation office conducts criminal investigations into illegal activities for FDA-regulated products such as food. Defendants may be arrested and punished for violation of the laws. The current research also suggests that while organizations may lack sufficient time and money to train employees about potential organizational wrongdoing or ethics violations and to have employees serve on ethics oversight or review boards, active mentoring is deemed necessary [35].

In addition, employees are participants in the operations of the stores and are forced to participate in the breach when the stores engage in food-handling practices that are inconsistent with legal requirements. As a result, regulatory agencies (government regulators and platforms) come more frequently to advise, the more employees fear that their indulgence in food safety violations will be perceived as adultery when the scandal comes to light and that they will be punished in the same way for it. In turn, this thinking will motivate them to proactively blow the whistle. We, therefore, have Hypothesis 2.

**H2.**  *The framing of Public-private governance systems positively affects employees’ intention to blow their whistle due to the risk to food safety.*

### 2.3. Employees’ Knowledge

Employees’ knowledge refers to their understanding of food safety incidents in their restaurants and their mastery of the pathway to participation in the public-private governance system, which is specifically evidenced by their familiarity with the policy of whistleblowing. Perceived behavior control(PBC) refers to people’s perception of the difficulty of performing a particular behavior, and it reflects employees’ judgment of the difficulty of reporting. Given that reporting a violation is risky behavior only if one is well-versed in reporting channels, a whistleblower may have sufficient confidence in reporting procedures and investigation procedures to make a report [32]. Familiarity with reporting channels is thus a key factor influencing employees to report. Several studies have confirmed that training and education programs are highly significant determinants and have been found to have a positive effect on whistleblowing. The skills and knowledge that whistleblowers gain through education and training programs increase the likelihood that they will actually blow the whistle and reduce the stress and limitations associated with the natural discomfort that whistleblowers feel [36]. Furthermore, in cases where the perceived personal cost of whistleblowing increases and the willingness to report decreases, education about whistleblowing may be a good way to dissipate this effect and lead to an increase in willingness to report [37].

Food security is a highly specialized task requiring a broad understanding of relevant knowledge. Food production, on the other hand, is a dynamic process, and risks at any step in the process can be carried forward to the next step. Thus, the allocation of responsibility for food safety risks faces challenges. Where it is easier to assign responsibility for food safety liability risks, the reported behavior can be dealt with quickly. Since the preparation of takeaway foods is a non-standardized process, the allocation of responsibility for food safety risks will be more challenging, i.e., the reporting of food safety risks may not always lead to the actual responsible party. Thus, the difficulty of attributing responsibility for food safety risks will also influence employees’ willingness to report, so we have Hypothesis 3 based on the above two points.

**H3a.**  *The employee who is more familiar with whistling channels has a greater intention to report food safety risks.*

**H3b.**  *Awareness of food safety violations positively affects employees’ intentions to blow the whistle.*

## 3. Data and Measurements

### 3.1. Data

A stratified sample of registered merchants from 16 different districts of Shanghai was selected for the study, divided into districts, and structured face-to-face interviews were conducted over a period of five months. Participants were aged 18 years or over and had worked in restaurants across a range of different employment divisions. Having stated the aims of our study, we proposed a protocol whereby their identities would be strictly anonymous. Simultaneously, participants in the post-survey condition received monetary incentives. Questionnaires were collected from 405 individuals at the end of the survey, and the average length of interview per respondent was 24 min. This study, after removing questionnaires with a response time shorter than 20 min, ultimately yielded 336 valid questionnaires for subsequent data analysis.

### 3.2. Measurements-Questionnaire Design

The questionnaire was designed on a research hypothesis framework with the exception of the personal details of the respondents (demographic data). The full contents of the questionnaire will be presented in Appendix A.

Given that previous research has found that individuals’ trust in regulators affects their attitudes toward whistleblowing, and potential whistleblowers may have their whistleblower excitement dulled by distrust of the government (including but not limited to the question of whether the government can operate effectively, if whistle-blowers are treated equally, or if the anonymity of their own private information can be protected). For example, an earlier study conducted a national survey in the Republic of Armenia and found that perceptions of a more trustworthy government had a positive influence on citizens’ attitudes toward whistleblowing [38]. For this reason, the present study also used a 5-point Likert scale to measure the perceived validity of subjects’ perceptions of food safety regulations (1 = completely disagree; 2 = disagree; 3 = neutral; 4 = agree; 5 = completely agree). Here are a few examples of items, “I think government governance of takeaway food safety is very effective” and “I think platforms are very effective in managing takeaway food safety”.

Prospective whistleblowers often worry about the damage to their reputation and money if their reports come to light, which may negatively influence their willingness to report, but Gundlach et al. found that mechanisms and procedures to protect whistleblowers’ identities and a clear set of procedural steps can foster whistleblowing behavior [39]. Furthermore, whistleblowing laws and procedures alone are not enough, and Smith et al. have further found through their research that education and training are one of the most important ways to promote a whistleblowing culture [40]. Thus, our study builds modestly on the survey methodology cited by previous authors and uses two indicators to measure the orientation of government and online platforms to restaurants. The first question asked was “whether government authorities guide restaurants on the safety of takeaway foods through advice, education, and training (1 = yes; 2 = no)”. The second question asked about participants’ perceptions of the online platform, “Does the online platform guide restaurants on the safety of takeaway foods through tips, education, training, and other measures (1 = yes; 2 = no).” Employees’ knowledge of food safety was measured using a question, “I can easily delineate the liability and sources of the food safety risks (1 = yes; 2 = no).” An additional question was used to measure respondents’ familiarity with the reporting channels, i.e., “I am familiar with the food safety reporting system currently being rewarded in Shanghai and China (1 = yes; 2 = no)”.

The behavioral intention was measured using a question that asked “If you found a food safety issue in a restaurant, would you be likely to report it to the system of governance? (1 = definitely yes; 2 = possibly, depending on the situation; 3 = definitely no)”. Finally, four items were selected for the individual characteristic variables that were (I1) gender (1 = male; 2 = female), (I2) Age, (I3) education (1 = primary school and below; 2 = junior middle school; 3 = technical high school/senior high school; 4 = college; 5 = undergraduate course; 6 = Graduate student), (I4) Position or status(1 = chef; 2 = server; 3 = cashier; 4 = delivery staff; 5 = others), (I5) monthly income(1 = less after tax than 3000 yuan; 2 = 3001–6000 yuan; 3 = 6001–12,000 yuan; 4 = 12,001–20,000 yuan; 5 = more than 20,000 yuan).

## 4. Empirical Results

### 4.1. Respondents

Our article starts with descriptive statistics, which give basic information about the respondents. The baseline characteristics of the respondents are shown in Table 1. A total of 46.43% of the 336 valid respondents and 53.57% were men and women, respectively, which is a moderate proportion and a reasonable selection of the sample, which is also roughly consistent with the true ratio of more females than males in Shanghai’s restaurant industry overall [41]. On the one hand, this result is due to the active tendency of firms to recruit: women are seen as more patient and caring than men, and suitable for service sector work. On the other hand, there is also the concept of Chinese history for thousands of years, according to which servers should be female. So many companies are consciously recruiting women as well. A total of 86.61% of respondents are foreign, which is consistent with migrant employees tending to opt for low-skilled and labor-intensive occupations. The age of respondents is concentrated between eighteen and thirty (70.84%) with the highest age being fifty. It is also consistent with the need for restaurant employees to fundamentally perform manual labor. Advantages of youth include good physical strength, listening to commands, and low pay. Furthermore, given that wages in the restaurant industry are typically not high, the majority of servers recruited are from rural or less economically developed areas. Many young people in rural areas leave their hometowns and enter the city to work and consider being a waiter to be their first priority. The educational backgrounds of the respondents are mainly concentrated in the years from junior high school to university. The remaining 35% are in high school or secondary school, which is closely related to the aforementioned personnel to rural, young, and small resources, in addition to the traditional concept of many people that the catering industry is not required to have a university degree, the industry has a low threshold of entry. Finally, more than half of the positions or identities in the food service industry are servers (55.36%), which is in line with the distribution of staff in actual positions in the restaurant industry. Overall, then, the sample has good randomness and can be expected to have a good representation as well as good coverage.

Meanwhile, by sorting the distribution of the results of the independent variables, we found that the mean values of the food industry insiders’ assessments of the effectiveness of food regulation by the government and takeaway food platforms were 2.23 and 2.38(Table 2), respectively (1 represents the most negative evaluation and 5 represents the most positive evaluation). respectively (1 represents the most negative evaluation and 5 represents the most positive evaluation). With an overall average level and relatively concentrated distribution. The average values of the whistleblowers’ understanding of the whistleblower system and the difficulty in delineating responsibility were 3.18 and 2.74, respectively, respectively (1 represents the most difficult and 5 represents the easiest) indicating that the sample provided moderate feedback regarding the level of understanding of Shanghai’s whistleblower system and the difficulty in determining liability for food safety violations. In conclusion, more than half of government and take-home platforms require businesses to guide takeaway food safety production and operation practices online through consultation, education, and training. However, the take-home platform’s guidance coverage is lower than the government’s and still needs to be improved.

### 4.2. Regression Results

Since the dependent variable of the study, whether or not to report, is a categorical variable and there are three levels of this variable (more than two), we conducted regression analysis using Multiple Ordered Regression Models. This study was then analyzed using stepwise regression. The valid data obtained in this study was put into SPSS software to perform the regression analysis. Model 1 placed only individual characteristics as independent variables, and Model 2 added to this the determinants of behavioral attitudes, i.e., perceived attitudes towards government and the management of takeaway platforms respectively. Model 3 then added subjective normative variables after controlling for individual characteristics and behavioral attitude variables, i.e., whether there is a government and platform orientation. Lastly, in Model 4, the four categories of variables were included as independent variables in order to explore their effects on employees’ willingness to blow the whistle. The significance of the model fit is less than 0.05, which means that at least one of the independent variables is valid and the model set is significant. Despite the fact that the pseudo-R-squared data were smaller than the expected value, the model was still deemed significant based on the degree of model fit in this study because the dependent variable in the model was a categorical variable. All models passed the parallel lines test (all *p* values were greater than 0.05, indicating that the dependent variables were ranked at the same level and that the parameter estimation table was valid). Final regression results were displayed in Table 3.

Firstly, it is found in Model 2 that an individual’s perceived effectiveness of the public governance system has a significant positive impact on their willingness to whistle blow food safety violations (B = 0.488, *p* < 0.01), while an individual’s perceived effectiveness of private governance has no impact on individual’s willingness to report food safety violations (B = 0.364, *p* > 0.05). In other words, as an indicator affecting individual behavior and attitude, government food monitoring may have a greater impact on food industry insiders’ reporting behavior, and the higher the subjects’ valuation of the government’s monitoring effect, the more likely they will choose to report when confronted with issues of food quality and safety. During the period of the epidemic, the central government introduced a series of regulatory activities aimed at safeguarding food safety—with the most stringent enforcement and accountability—and increased its efforts to crack down on food safety violations, which greatly mitigated citizen concerns and further deepened the individual’s trust in government oversight. There is no doubt that this increases the whistle-blower’s confidence in the positive outcome of their reporting. At the same time, in order to prevent and control the epidemic effectively, home quarantine has become a reality, but the resulting “quarantine economy” has provided new opportunities for development in the takeaway industry. In the short term, takeaway food has become an important steppingstone for restaurant businesses to survive the epidemic crisis. The epidemic has, in the long run, accelerated the development of the food service industry to some extent, where the combination of online and offline will become the new normal for the development of China’s food service industry. During the epidemic, takeaway showed great superiority, and it brought new changes and vitality to the entire industry. However, because of the surge in orders, online take-home platforms do not have sufficient incentive to monitor the compliance of platform riders and merchants in a timely fashion. Consequently, there are various superficial “preventative checks” that prevent potential internal whistleblowers from having sufficient confidence and trust in the take-home platform, and further challenge the legitimacy of their control.

Second, government guidance to food industry merchants was found to have no effect on employees’ willingness to report (B = −0.065, *p* > 0.05) in Model 3. Contrary to the research hypothesis, advice from the online take-home platform to delivery merchants inhibits food industry employees’ willingness to report (B = −0.777, *p* < 0.01). That is, if the take-home platform has guiding behavior for market traders, store staff will be reluctant to report after food safety violations are discovered. On the other hand, whether or not the government engages in guiding behavior has no impact on its intentions to blow the whistle. China’s system of food safety regulation was established later, compared to developed Western countries, but since the enactment of the Food Safety Law of the People’s Republic of China in 2009, China’s system of food safety regulation has been subject to continuous improvement. With the continuous development of China’s socialist market economy and the improvement of people’s living standards, there has been a gradual shift in people’s demand for food from ‘eating enough’ to ‘eating well’. Online food ordering and offline food mode delivery also due to their convenient and affordable features become the main groups of food ordering habits. The food market environment becomes increasingly complex and unstable in such a transformative process, and the regulatory authorities’ static (or this is to say slowly changing) approach to regulation is gradually becoming inconsistent with the development trend as a whole. With the advent of the epidemic, such regulatory gaps have been amplified. Meanwhile, the take-home platform’s guidance to merchants itself lacks some legal foundation and coupled with the rapid development of the “isolation economy” brought about by the epidemic, the platform, profit-driven, has gradually slackened its supervision of compliant operations. The consequence of this is lax management (e.g., lack of monitoring of rider body temperature and failure to wear a mask cannot be effectively detected and stopped in a timely way), as a result, the take-home platform ends up losing the trust of merchants and the incentive effect of employee guidance on them is counterproductive.

Lastly, Model 4 demonstrated that individuals’ knowledge of the local or national reporting system had a significant positive effect on the intentions of food initiates to report (B = 0.318, *p* < 0.05), meaning that the more knowledgeable they were about the reporting system, the stronger their intentions to report violations of the food safety system. The level of significance for the relationship between difficulty assigning responsibility and a person’s willingness to report is also greater than 0.05. Therefore, it is reasonable to believe that the difficulty of assigning responsibility for food safety violations does not influence the ultimate decision of insiders in the food industry when deciding whether or not to disclose a food safety violation. It is crucial to concede that the sudden COVID-19 outbreak disrupted the pace of everyone’s lives, work, and studies. The outbreak has made food safety a top priority. In order to strictly enforce the ‘four strictest’ requirements and to effectively protect the health and safety of people’s lives, cities across the nation have relied on websites, newspapers, WeChat, Weibo, TikTok, SMS, and other platforms to vigorously promote food safety knowledge, in an effort to increase public awareness of and satisfaction with food safety issues. The data collected in this article suggest that this has proven beneficial in reducing barriers on the road to employee reporting.

## 5. Discussions and Managerial Overviews

### 5.1. Discussion

The study found that the perceived effectiveness of the public governance system and employees’ knowledge of the food safety reporting system had a significant positive effect on the willingness of whistle-blowers to report food safety violations, whereas guidance from the online take-home platform significantly inhibited their willingness to self-report. At the same time, employees’ perceived effectiveness of the private governance system (the takeaway platform), the guiding behavior of the government, and the difficulty of apportioning responsibility for food safety violations did not influence the intentions of food safety insiders to blow the whistle.

The ideology of “king as dynasty” that is prevalent in China is a plausible explanation for the fact that employees’ perceived effectiveness of the public governance system may significantly influence their willingness to report a violation of food security once they are identified. For individuals, the government is an authority, which means that government regulatory actions are perceived by employees as justified and legitimate, and thus it is only the perceived effectiveness of government oversight that may lead individuals to have a positive or negative attitude towards whistleblowing behavior. Furthermore, individuals’ evaluations of the effectiveness of government food safety regulations further reflect their endorsement and confidence in the government’s work. They believe that the higher the level of endorsement and trust, the more likely it is that the government will be able to handle their reported information correctly and reasonably, and the greater their expectation that reporting behavior can produce the desired effect. During the period of epidemic prevention and control, the Chinese government introduced a series of measures aimed at strengthening the regulation of imported cold chain fresh foods, which achieved remarkable results in the prevention and control of COVID-19 importation from abroad and the food safety of consumers. There is no doubt that these actions have deepened people’s trust in the government and recognition of its regulatory capabilities. This finding is consistent with the findings of Du et al., who concluded that institutional trust could have a positive influence on individuals’ willingness to report [17]. In the case of take-home platforms, individuals’ willingness to blow the whistle has not been influenced by their perceived efficacy of the private governance system. One possible reason for this is that cross-platform competition has increased as more and more take-home platforms have been created. Platforms will choose to form alliances with traders in order to attract more traders to enter to maximize their own profits. The platform will then intentionally turn a blind eye to the marketer’s unregulated production and exploitation of food. Based on the Shanghai Food and Drug Administration’s ten consecutive rounds of online food service surveillance, it was found that many online merchants engaged in illegal behavior during monitoring [42]. However, after being notified by the platform, it was discovered that these merchants were once again online in the follow-up surveillance, which demonstrates that the platform is simply not performing the primary responsibility well. In the existing study, Garcia et al. emphasize that private and social interests are often distinct and that from the point of view of private companies, an effective food safety control system may not produce socially beneficial outcomes, leaving them without an incentive to actively monitor [14]. Therefore, this implies that insiders in the food industry do not trust this platform’s ability to effectively regulate food safety. The loose management of dealers and delivery staff by online take-home platforms (e.g., daily temperature measurement with no monitoring mechanism; unregulated mask-wearing) makes practitioners skeptical of the platform’s monitoring effectiveness, and that vague rating may not manipulate or influence their willingness to disclose.

Contrary to expectations, Government and platform guidance had unforeseen outcomes. The direction of the platform has a suppressing effect on the willingness of internal employees to blow the whistle, which is in contrast to the study hypothesis. Upon detailed investigation, however, this result is also within the realm of explanation. The influence of COVID-19 has accelerated the development of online take-home platforms, which are already in a rapid stage of development, and adopted a second spring. A vendor’s market with its own unique management system and industry ethos has been formed by a group of leading platform operators, and the merchants on the platform are obliged to follow these formed ‘rules’, either explicitly or implicitly [43]. However, since the take-home platform is always driven by profit, the sense of self-restraint is not enough, the operating system is not standardized, and it may even go as far as to touch the red line of the law. This, in turn, has led to the formation of a coalition with non-compliant companies [44], which artificially creates factors of unsafe food production. For this reason, insiders in the food industry are always wary of platform management behavior. The distrust and stereotyping of online take-home platforms suggest that individuals may perceive the guiding behavior of the platforms as a threat, leading to a rebellious mentality [45]. The sociological explanation suggests that rebellion may occur when coercive and absolute demands arise and constrain people’s choices, with the immediate consequence that any persuasive point of view will be struck down as a threat to freedom [46,47]. Furthermore, from the employee’s point of view, the relationship between the online take-home platform and the marketer is based solely on cooperation, which is reflected in the payment of fees for platform management in order to get online sales channels. Thus, from the employee’s point of view, the online platform lacks legitimacy in guiding and regulating the marketer. Studies have shown that platforms differ in their effectiveness when the government identifies food security risks with different likelihoods. It is difficult to see how increased platform supervision alone can fully play a role in deterring corporate violations. Rather, multistakeholder coordination is the best mode of monitoring [16]. In terms of why the government’s referral behavior did not impact willingness to report, this can also be explained using reverse psychology. Individuals’ acceptance of stimuli is limited, and moderate stimuli have the potential to stimulate personal development and bring great satisfaction. When the stimulus is excessive, it becomes a sort of pressure or even a detriment to the individual and the individual will take evasive action [48]. In contrast to the perceived effectiveness of government food safety regulation, companies must passively accept government directives, including, but not limited to, the popularization of food safety laws, regulations, food safety standards, and daily quarantine requirements, in order to promote a healthy diet and increase people’s awareness of food safety. As part of this process, in order to reach a larger group of companies in a shorter period of time, inevitably, the government will have a mere repetition of content and forcible indoctrination. Particularly in the case of inadequate manpower in epidemic areas, mechanistic and process-oriented indoctrination of standards does not allow food practitioners to develop subjective standards but instead causes them to become numb or even overwhelmed in response to such stimuli. This finding is consistent with Yin et al. (2017) that government guidance has a slight influence on the public’s willingness to conduct whistleblowing actions [49,50].

We conclude that personal knowledge of the local food safety reward notification system had a significant positive impact on intentions to blow the whistle on the employee. However, the difficulty of dividing authority and responsibility had no effect on it. This may be due to the fact that the subjects of this paper are predominantly bottom-up employees, which means that the personal cost of whistleblowing behavior is much lower than that of those in other senior positions or positions. Consequently, the probability of retaliation and loss of one’s job in the face of a whistleblowing dilemma is also lower, in addition to the relatively high accessibility of finding alternative similar jobs. In the case of food quality and safety violations, the lower ranks mean that they have little pressure to bear the consequences of their mistakes, thus, the degree of difficulty in dividing specific authority and responsibility does not come at a material cost to them after reporting, thus rendering this indicator substantively unrelated to employees’ willingness to report. Their priority is whether or not they can be protected from being challenged by the regulator’s sanction. Of course, if their whistleblowing does not hurt them, they will be happy to report and even have the opportunity to be rewarded for it. On the other hand, the level of knowledge about the whistleblowing system—including the reporting process, the criteria for reporting, the rewards for reporting, and the potential evidence that needs to be provided at the time of reporting—restricts the control of potential whistleblowers have over their reporting behavior. Thus, the higher the level of knowledge about the whistleblowing system, the more confident individuals are in achieving the desired outcome of their whistleblowing behavior and are more likely to be pressured to engage in whistleblowing. For example, consider an online famous bakery based in Shanghai. The bakery’s employees exposed on Weibo the problems of oil, rodent infestation, and expired flour in the operating room of the bakery. The bakery did have food safety hazards, after verification by the market oversight department. It is difficult to determine in this case who is responsible for the illicit operation of the bakery, and the difficulty of apportioning responsibility between the Public Security Bureau and the Labor Bureau. However, this did not affect the whistle-blower’s judgment in carrying out the report. He makes his report based on his knowledge of policies related to whistleblowing, and whistleblowing ultimately helps the government to conduct investigations.

### 5.2. Management Applications

Given that individual trust in regulatory topics (including recognition of the capacity to deal with problems, protection of whistleblower information, etc.) largely shapes employees’ willingness to blow the whistle, during the COVID-19 prevention phase, the government should take full advantage of the positive effects of the regulatory results to guide whistleblowers to bravely point out food quality issues, thereby reducing governance costs and forming a virtuous cycle. Some studies have shown that government measures and feedback incentives play an important role in increasing the public’s motivation to participate in pro-social behavior [50]. Thus, a feedback mechanism for food safety reporting may help to guide potential whistleblowers to develop a positive attitude towards food safety reporting and to increase their awareness of being involved in food safety reporting. On the other hand, because take-home platforms, as systems of private governance, are in a state of disbelief in the minds of individuals, to better constrain their day-to-day operations, the government should endeavor to reverse the situation by imposing tougher penalties on take-home platforms for violations. At the same time, the platform should also proceed on its own to build the confidence and trust of companies with a standardized management model by clarifying the entry requirements for online catering companies.

The distrust of the platform also makes the employees rebel badly against the platform’s direction, which the government does not want to see. The reason for this is that it is extremely necessary to use the power of online take-home platforms when a violation of food safety is difficult and costly to achieve the desired effect through centralized governance by government departments alone. for the platform to play its role, as noted above, it is crucial to reverse employees’ mistrust of it. First, take-home platform operations should be restricted and those that are poorly regulated should be notified or added to the “blacklist”, or even (as in the case of platforms that cause serious adverse consequences) for the purpose of imposing administrative sanctions, in an effort to gradually change food industry employees’ distrusted attitudes toward the platform. This increases the platform’s cost of illegality, meaning that it provides food industry insiders with a reason to trust the platform’s image of its integrity. Then it is recommended that the government implement a strategy of “empowerment” in order to give the platform the corresponding legitimacy of management and oversight, particularly in the form of clarifying take-home platform responsibilities and obligations. This allows the government to exchange information with platforms and improve the effectiveness of their implementation. Moreover, in response to the embarrassing situation of the merchants’ indifference to government advice, the government should also actively make changes after finding the key to breaking the problem, such as the adoption of softer, more diverse food safety training resources with a focus on the serious consequences of unregulated operations (e.g., the cunning nature and high spread of viruses), and the avoidance of a single repetitive and tedious formalistic problem. In general, the government should provide the platform with greater legitimacy for food quality and safety governance and apply more responsive food safety training resources to decrease the mental burden of passive education.

The findings of the study suggest that the diffusion and popularization of whistleblowing policies and systems are critical. Since the whistleblower system in China is not well-established at this time and even fewer studies of whistleblowers in epidemic prevention and prevention. The need for rapid development and improvement of China’s epidemic whistleblower system is evident. The particular nature of the epidemic highlights the importance of improving and promoting a rewarding whistleblowing system and strengthening the rewards for whistleblowers. The reason for this is that whistleblower status requires not only a sense of justice, conscientiousness, and courage, but also material and spiritual encouragement and support. The rule-of-law economic system allows the whistleblower system to achieve double the outcome with half the effort compared to the governance effect with a large amount of government spending. Furthermore, in the event of an emergency or a sudden outbreak, there should be a break in the regular administrative processes, and the need for government to implement and process whistleblowing information in a timely and rapid manner. An important reason for this is that COVID-19 is distinct from ordinary food quality questions. The public interest, if the report is true, will cause widespread disruption in a very short amount of time. On the other hand, if Regulators Fail To Respond in a timely manner, in extreme cases, we cannot avoid the fact that reporters of outbreaks will choose to expose themselves to the media or make them public online. Given that the epidemic is such a sensitive subject in those years, there is a high likelihood that it will spread so widely with heated discussions that have the potential to cause public panic and further threaten national security.

### 5.3. Conclusions

COVID-19 has become a common global challenge, with countries around the world, including China, investing significant resources in outbreak prevention and control. At the same time, the new coronavirus epidemic prevention and control work involves all walks of life, social and economic aspects, the food industry is no exception. With the development of the epidemic, the emergency protection function and future strategic reserve function of the food industry come to the fore. While the virus has not been enough to prompt countries to take overly vigilant regulatory measures, such as preventing food from flowing from hard-hit regions to other regions, food hygiene requirements have increased dramatically across all food manufacturing industries, including manufacturers, retailers, and restaurants, putting more pressure on China’s food safety governance.

Collaborative public-private governance is widely believed to be a good way to reduce costs and improve the effectiveness of government regulations. The majority of research has focused on studies of government regulation and the resultant consumer satisfaction and trust in current government regulation of food security and the factors that influence it. A limited amount of literature has examined food safety governance from a food safety blowback perspective, and the majority of the limited studies in the literature focus on theoretical countermeasure studies, with a paucity of literature examining employee food safety reporting with field research and few empirical studies reported on factors influencing employees’ willingness to whistle blow food safety violations in China. Consequently, this paper studies the willingness-to-participation of public-private collaborative governance by whistleblowing China’s food safety whistleblowers, using data from field research in Shanghai as an example. The study offers recommendations for the public-private governance system, which promotes a shift from administrative control to public governance.

Although this study explores the underlying reasons for employees’ participation intention in food safety regulation by whistleblowing, some extensions are able to be conducted in the future. First, the data in this study is surveyed in Shanghai. Shanghai is one of the most prosperous areas in China, some research should focus on more small cities and rural areas which might result in diverse research findings. Second, this study presents the determinators based on the perspective of planned behavior theory. However, individuals sometimes are irrational in making decisions; an employee’s whistleblowing behavior is partly determined by many factors, such as emotions to food safety volitation, and recognition of food safety. Thus, more factors are able to be considered for more extensions.

## Figures and Tables

**Table 1:** Sociodemographic Characteristics of Respondents.

N = 336	Components	Frequency	Percentage
Gender	Male	156	46.43%
	Female	180	53.57%
Hukou	Local	45	13.39%
	Not local	291	86.61%
Education level	Primary school and below	6	1.79%
	junior middle school	90	26.79%
	Technical secondary school/high school	149	44.35%
	college	69	20.54%
	undergraduate course	21	6.25%
	Graduate student	1	0.30%
Age(years)	Under 20	64	19.05%
	21–30 Years old	174	51.79%
	31–40 Years old	74	22.02%
	41 and above	24	7.14%
Position	server	186	55.36%
	cashier	66	19.64%
	delivery personnel	13	3.87%
	chef	71	21.13%

**Table 2:** Descriptive statistics of explanatory variables.

Variable	Variable	Components	Frequency	Percentage
Perceived effectiveness of governance system	Perceived effectiveness on Government	Effect evaluation of government supervision	2.23	0.862
	Perceived effectiveness on Platform	Effect evaluation of the supervision of the takeaway platform	2.38	0.823
Guidance from governance system	Government’s Guidance	Guidance from government	248	73.80%
	Platform’s guidance	Guidance from online platform	223	66.40%
Knowledge	Knowledge of system	The knowledge of whistle system	3.18	0.915
	Knowledge of risk	The knowledge of food safety risk	2.74	0.818

**Table 3:** Multiple Ordered Regression Models.

Variables	Model 1		Model 2		Model 3		Model 4		VIF
	B	Wald	B	Wald	B	Wald	B	Wald	
Gender (male)	−0.350 (0.256)	1.877	−0.411 (0.266)	2.394	−0.467 (0.270)	2.999	−0.422 (0.273)	2.391	1.143
Age	−0.003 (0.015)	0.050	0.001 (0.016)	0.003	−0.005 (0.016)	0.105	−0.004 (0.016)	0.055	1.125
Education	0.114 (0.132)	0.741	0.076 (0.136)	0.316	0.055 (0.137)	0.163	0.077 (0.139)	0.304	1.164
	−0.350 (0.256)	1.877	−0.411 (0.266)	2.394	−0.467 (0.270)	2.999	−0.422 (0.273)	2.391	
Monthly income	0.378 (0.209)	3.265	0.236 (0.220)	1.147	0.282 (0.222)	1.611	0.240 (0.224)	1.150	1.131
Position (server)	0.285 (0.300)	0.901	0.292 (0.310)	0.888	0.226 (0.315)	0.514	0.246 (0.318)	0.599	1.098
Position (cashier)	0.389 (0.393)	0.982	0.400 (0.405)	0.976	0.430 (0.408)	1.111	0.410 (0.410)	1.000	
Position (delivery personnel)	−0.277 (0.605)	0.210	0.201 (0.627)	0.102	−0.173 (0.632)	0.075	−0.127 (0.642)	0.039	
Perceived Effectiveness of Government			0.488 ** (0.162)	9.061	0.457 ** (0.167)	7.529	0.429 * (0.168)	6.524	1.477
	
Perceived Effectiveness of Platform			0.364 (0.173)	4.420	0.263 (0.178)	2.187	0.217 (0.181)	1.425	1.545
	
Government Guidance					−0.065 (0.307)	0.045	0.019 (0.309)	0.004	1.300
			
Platform guidance					−0.777 ** (0.296)	6.897	−0.729 * (0.296)	6.058	1.287
			
Knowledge of system							0.318 * (0.138)	5.288	1.132
Knowledge of risk							−0.020 (0.147)	0.019	1.035
Parallel line	0.113		0.194		0.236		0.243		
Pseudo-R square	0.017		0.099		0.122		0.136		

Note: Standard errors in parentheses. * *p* < 0.05; ** *p* < 0.01; two-tailed test.

## Data Availability

The data presented in this study are available on request from the corresponding author.

## Appendix A

**Table A1:** Questionnaire.

Gender	1 = Male; 2 Female
Hukou	Local = 1; Not local = 2
Age	Under 20 = 1; 21–30 = 2; 31–40 =3; 41 and above = 4
Education	Primary school and below = 1; junior middle school = 2; Technical secondary school/high school = 3; undergraduate = 4; Graduate and above = 5
Position	Server = 1; cashier = 2; delivery = 3; chef = 4
Salary	3000 Yuan and less = 1; 3001–6000 Yuan = 2; 6001–12,000 Yuan = 4; 12,001–20,000 = 4; More than 20,000 = 5
Government Governance	I think government governance of takeaway food safety is very effective completely disagree = 1; disagree = 2; neutral = 3; agree = 4; completely agree = 5
Platform’s management	I think platforms are very effective in managing takeaway food safety. completely disagree = 1; disagree = 2; neutral = 3; agree = 4; completely agree = 5
Government guidance	Have any government authorities guided your restaurants on the safety of takeaway foods through advice, education, and training Yes = 1; No = 2
Online platforms’ guidance	Have the online platforms guided your restaurants on the safety of takeaway foods through tips, education, training, and other measures Yes = 1; No = 2
Knowledge of food risk	I can easily delineate the liability and sources of the food safety risks Yes = 1; No = 2
Knowledge of whistleblowing system	I am familiar with the food safety whistleblowing channels in Shanghai and China Yes = 1; No = 2
Whistleblowing intention	If you found a food safety issue in a restaurant, would you be likely to report it to the system of governance? Yes = 1; No = 2
